# Artificial intelligence for prostate MRI: open datasets, available applications, and grand challenges

**DOI:** 10.1186/s41747-022-00288-8

**Published:** 2022-08-01

**Authors:** Mohammed R. S. Sunoqrot, Anindo Saha, Matin Hosseinzadeh, Mattijs Elschot, Henkjan Huisman

**Affiliations:** 1 Department of Circulation and Medical Imaging, NTNU–Norwegian University of Science and Technology, 7030 Trondheim, Norway; 2 Department of Radiology and Nuclear Medicine, St. Olavs Hospital, Trondheim University Hospital, 7030 Trondheim, Norway; 3 Diagnostic Image Analysis Group, Department of Medical Imaging, Radboud University Medical Center, Nijmegen, 6525 GA The Netherlands

**Keywords:** Artificial intelligence, Deep learning, Image processing (computer-assisted), Multiparametric magnetic resonance imaging, Prostatic neoplasms

## Abstract

Artificial intelligence (AI) for prostate magnetic resonance imaging (MRI) is starting to play a clinical role for prostate cancer (PCa) patients. AI-assisted reading is feasible, allowing workflow reduction. A total of 3,369 multi-vendor prostate MRI cases are available in open datasets, acquired from 2003 to 2021 in Europe or USA at 3 T (*n* = 3,018; 89.6%) or 1.5 T (*n* = 296; 8.8%), 346 cases scanned with endorectal coil (10.3%), 3,023 (89.7%) with phased-array surface coils; 412 collected for anatomical segmentation tasks, 3,096 for PCa detection/classification; for 2,240 cases lesions delineation is available and 56 cases have matching histopathologic images; for 2,620 cases the PSA level is provided; the total size of all open datasets amounts to approximately 253 GB. Of note, quality of annotations provided per dataset highly differ and attention must be paid when using these datasets (*e.g.,* data overlap). Seven grand challenges and commercial applications from eleven vendors are here considered. Few small studies provided prospective validation. More work is needed, in particular validation on large-scale multi-institutional, well-curated public datasets to test general applicability. Moreover, AI needs to be explored for clinical stages other than detection/characterization (*e.g.*, follow-up, prognosis, interventions, and focal treatment).

## Key points

- Artificial intelligence shows promise for being applied to prostate cancer magnetic resonance imaging (MRI).
- Open datasets for prostate MRI are limited.
- Commercial solutions are available but lack adequate validation.
- Grand challenges could provide the means for bias-free validation.

## Background

Prostate cancer (PCa) is the second most prevalent cancer among men worldwide [1]. Nevertheless, the mortality rate is relatively low, and most patients die with and not of PCa [2]. Timely and accurate diagnosis is therefore of utmost importance to avoid overtreatment of men with indolent, clinically insignificant PCa, and to offer radical curative treatment to men with life-threatening, clinically significant PCa (csPCa) [3]. Present-day guidelines advise the use of multiparametric magnetic resonance imaging (mpMRI) prior to biopsies [3], as it can noninvasively discriminate patients with indolent PCa from those with csPCa, retaining a high sensitivity for csPCa [4–6]. Using the version 2 of the Prostate Imaging Reporting and Data System (PI-RADS) [7], radiologists make a semiquantitative assessment of each suspicious lesion observed on mpMRI and assign a corresponding csPCa likelihood score, from 1 to 5. Together with clinical variables, such as patient age, prostate-specific antigen (PSA) levels, and family history, PI-RADS scores help clinicians determine whether further investigation (via systematic or targeted biopsies) is needed to make a final diagnosis.

**Fig. 1:**
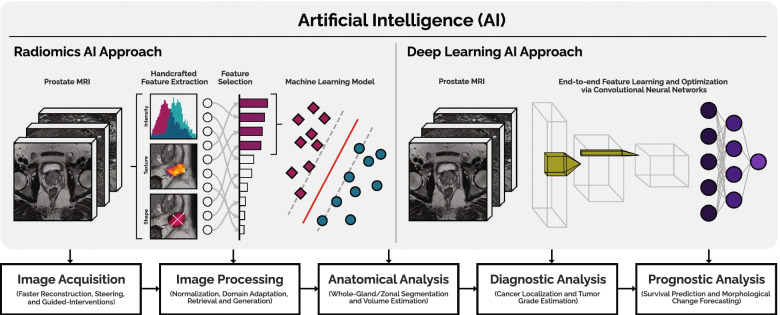
Use of artificial intelligence in the radiological workflow of prostate magnetic resonance imaging to automate, improve, and support critical tasks, considering radiomics and deep learning approaches

At present time, the processing and interpretation of prostate mpMRI data in clinical routine is entirely performed by human experts (radiologists) who, while competent, are time-limited, cost-intensive, and cannot be easily scaled to meet increasing imaging demands [8]. Furthermore, human performance is dependent on experience and training, leading to significant variability between observers [9–11]. In contrast to purely qualitative interpretation, artificial intelligence (AI) exploits the quantitative nature of mpMRI data. AI can automate and support (parts of) the radiological workflow (Fig. 1), improve diagnostic accuracy, reduce costs, and alleviate the workload of healthcare personnel.

In recent years, continuous technical developments and increased dataset quantity and quality have pushed AI performance close to that of experienced radiologists [12–14], leading to the emergence of both publicly and commercially available solutions. However, adequate validation via large-scale retrospective multicenter studies or prospective clinical studies is often still lacking. To realize this, the prostate MRI community should invest in curating large-scale, multicenter datasets, develop a unified methodology for standardized performance estimation, reach consensus on the reference outcome standard (beginning from the presence/absence of csPCa), and establish the minimum requirements for potential testing cohorts.

The purpose of this narrative review is to provide an overview of open datasets, commercially/publicly available AI systems, and grand challenges for prostate mpMRI. We focus on methods for segmenting prostate anatomy, and for diagnosis and localization of csPCa. While the prostate segmentation can facilitate the calculation of PSA density (and also guide treatment planning and future interventions), diagnosis and localization can inform risk stratification and biopsy strategies. As we approach a new phase in AI applications to prostate mpMRI, where the goal is to move towards transparent validation and clinical translation, we specifically report studies that investigated commercially or publicly available AI systems. Furthermore, we summarize publicly available MRI data that can be used to accelerate the development of AI systems and discuss the increasingly important role of grand challenges, which allow for bias-free benchmarking of AI algorithms applied to prostate mpMRI.

### Open datasets

AI, especially deep learning, requires large, well-curated datasets to facilitate training and meaningful validation [15]. Furthermore, models require diverse, multicenter, multivendor data to achieve robust performance and generalization. However, most algorithms reported in literature thus far, use relatively small, single-center datasets [16]. The limited number and quality of publicly available datasets for prostate MRI, further aggravates this issue.

Table 1 provides an overview of 17 public datasets for prostate MRI, which were found by the authors through their collaborative role in this research field and was updated with additional internet searches (*i.e.,* The Cancer Imaging Archive, Zenodo, XNAT, GitHub, and grand-challenge.org). A total of 3,369 prostate MRI cases (including some overlapping cases) are available, of which 2,238 cases primarily include mpMRI images acquired between 2003–2021 in Europe and the United States. All cases were provided as full 3D volumes, except for the QUBIQ21 dataset [17], which provided a single slice per case. A total of 412 cases were collected for anatomical segmentation tasks, whereas the remainder were collected for PCa detection and/or classification. The majority of cases were scanned with a 3-T scanner, whereas only 296 cases were scanned with a 1.5-T scanner. Scanner vendors include the following: Siemens (Siemens Healthineers, Erlangen, Germany), Philips (Philips Healthcare, Best, The Netherlands), and GE (General Electric Healthcare Systems, Milwaukee, WI, USA) for 2571, 446, and 110 cases, respectively. A total of 346 cases were scanned with endorectal coils, whereas the remaining were scanned with phased-array surface coils. In 2,240 cases lesion delineations are available and 56 cases have matching histopathologic section images obtained from radical prostatectomy specimen. Table 1 shows that clinical variables are available for some cases, *e.g.,* 2,620 cases with an associated PSA level. The scans are available in Digital Imaging and COmmunications in Medicine (DICOM), ITK MetaImage or NIFTI format for 1,547, 1,580, and 242 cases, respectively. Total size of all open datasets (images, annotations, and meta-data) amounts to approximately 253 GB. In 2021, delineations of PCa lesions and prostatic zones for (parts of) the PROSTATEx dataset [18] were curated by an independent third-party and publicly released at [19].

**Table 1:** Summary of prostate MRI public datasets

Number	Dataset	Data source	Modalities	Dataset size	Acquisition years	Files size	Data type	Field strength	Scanner manufacturer and model	Coil type	Clinical variables	Purpose	Reference
1	PROSTATE-MRI	TNCI	MRI (triplanar T2W, DWI, pre-contrast T1W, DCE)	MRI cases (*n* = 26), pathology images (*n* = 26)	2008–2010	3.4 GB	MRI cases (DICOM), pathology images (JPEG)	3 T	Philips Achieva	Endorectal	Gleason grade (*n* = 26)	Disease classification	[20]
2	Prostate Fused-MRI-Pathology	WRUHC, HUP	MRI (triplanar T2W, DWI, pre-contrast T1W, DCE)	MRI cases (*n* = 28), pathology images (*n* = 16)	2009–2011	81.2 GB	MRI cases (DICOM), pathology images (TIFF, XML)	3 T	Siemens Verio	Endorectal	Not reported	Disease classification	[21]
3	Prostate-3T	RUMC	MRI (axial T2W)	MRI cases (*n* = 64), SV and NVB segmentations (*n* = 15)	2003–2005	610 MB	MRI cases (DICOM), SV and NVB segmentations (MHA)	3 T	Siemens Trio	Pelvic phased-array surface	Not reported	Cancer detection	[22]
4	ICCVB	Not reported	MRI (axialT2W, DWI, DCE, ADC)	MRI cases (*n* = 12)	Not reported	5.6 GB	MRI cases (DICOM)	1.5 T (*n* = 7), 3 T (*n* = 5)	GE (*n* = 7), Siemens (*n* = 5)	Endorectal (*n* = 7), phased-array surface (*n* = 5)	Not reported	Cancer detection	[23]
5	TCGA-PRAD	UPMC, WHSL, LHMC	MRI (axial T2W, DWI, pre-contrast T1W, DCE, ADC), CT, PET	MRI cases (*n* = 10), CT images (*n* = 4), PET images (*n* = 3), pathology images (*n* = 14), genomics data (*n* = 14)	Not reported	3.74 GB	MRI cases (DICOM), CT images (DICOM), PET images (DICOM), pathology images (WEB), genomics data (WEB)	1.5 T	GE Signa HDx	Endorectal	PSA (*n* = 14), Gleason grade (*n* = 14), lesion location (*n* = 14), TNM classification (*n* = 14)	Disease classification, Cancer detection	[24]
6	PROSTATE-DIAGNOSIS	BMC	MRI (axial and coronal T2W, DWI, pre-contrast T1W, DCE)	MRI cases (*n* = 92), SV and NVB segmentations (*n* = 15), zones and lesions segmentations (*n* = 5)	2008–2010	5.6 GB	MRI cases (DICOM), SV and NVB segmentations (MHA), zones and lesions segmentations (NRRD)	1.5 T	Philips Achieva	Endorectal	Full report for: MRI (*n* = 54), biopsy (*n* = 43), specimen (*n* = 25), treatment (*n* = 52)	Disease classification, Cancer detection	[25]
7	Prostate-MRI-US-Biopsy	UCCUC	MRI (axial T2W), Ultrasound	MRI cases (*n* = 842), ultrasound cases (*n* = 1,151), gland and lesions segmentations (*n* = 1,150)	2004–2011	78.2 GB	MRI cases (DICOM), ultrasound cases (DICOM), gland and lesions segmentations (STL)	MRI: 3 T (*n* = 807), 1.5 T (*n* = 35). Ultrasoun: 2–10 MHz	MRI: Siemens Skyra (*n* = 609), Siemens Vida (*n* = 60), Siemens Prisma (*n* = 38), Siemens Verio (*n* = 36), Siemens Trio (*n* = 32), Siemens Avanto (*n* = 31), Siemens Espree (*n* = 1), GE Discovery MR750w (*n* = 14), GE Signa HDx (*n* = 10), GE Optima MR450w (*n* = 2), Philips Achieva (*n* = 5), Philips Ingenia (*n* = 4). Ultrasound: Hitachi Hi-Vision, Noblus C41V	MRI: Transabdominal phased-array surface. Ultrasound: End-fire probe	PIRADS (*n* = 840), PSA (*n* = 1146), Gleason grade (*n* = 196), prostate volume (*n* = 1,150), lesion location (*n* = 1,150)	Disease classification, Cancer detection	[26]
8	QIN PROSTATE	BWH	MRI (axial T2W, DWI, pre-contrast T1W, DCE, ADC)	MRI cases (*n* = 22)	Not reported	4.4 GB	MRI cases (DICOM)	3 T	GE Signa HDx	Endorectal	Not reported	Disease classification, Cancer detection	[27]
9	QIN-PROSTATE-Repeatability	BWH	MRI (axial T2W, DWI, DCE, ADC)	MRI cases (*n* = 15) zones and lesions segmentations (*n* = 15)	2013–2015	14.86 GB	MRI cases (DICOM), zone and lesions segmentations (DICOM)	3 T	GE Signa HDx	Endorectal	Not reported	Repeatability measurements, Disease classification	[28]
10	SPIE-AAPM-NCI PROSTATEx Challenges	RUMC	MRI (triplanar T2W, DWI, DCE, ADC, PDW)	MRI cases (*n* = 346), Ktrans images (*n* = 345), thumbnails images (*n* = 345)	2012	15.4 GB	MRI cases (DICOM), Ktrans images (MHD), thumbnail images (BMP)	3 T	Siemens Trio (*n* = 57), Siemens Skyra (*n* = 289)	Pelvic phased-array surface	Lesion location (*n* = 344), significant/insignificant cancer (*n* = 204), Gleason grade group (*n* = 99)	Disease classification, Cancer detection	[18, 29]
11	NCI-ISBI 2013 ASPS Challenge	BMC, RUMC	MRI (axial T2W)	MRI cases (*n* = 80), zones segmentations (*n* = 80)	2003–2010	600 MB	MRI cases (DICOM), zones segmentations (NRRD)	1.5 T (*n* = 40), 3 T (*n* = 40)	Philips Achieva (*n* = 40), Siemens Trio (*n* = 40)	Endorectal (*n* = 40), pelvic phased-array surface (*n* = 40)	Not reported	Zones segmentation	[30, 31]
12	PROMISE12 Challenge	HUH, BIDMC, UCL, RUMC	MRI (axial T2W)	MRI cases (*n* = 80), whole gland segmentations (*n* = 50)	Not reported	1.2 GB	MRI cases (MHD), gland segmentations (MHD)	1.5 T (*n* = 30), 3 T (*n* = 50)	GE (*n* = 20), Semiens (*n* = 60)	Endorectal (*n* = 41), phased-array surface (*n* = 39)	Not reported	Whole gland segmentation	[32, 33]
13	Medical Segmentation Decathlon	RUMC	MRI (axial T2W, ADC)	MRI cases (*n* = 48), zones segmentations (*n* =32)	Not reported	229 MB	MRI cases (NII.GZ), zones segmentations (NII.GZ)	3 T	Not reported	Phased-array surface coil	Not reported	Zones segmentation	[34, 35]
14	QUBIQ21 Challenge	Not reported	MRI (axial T2W)	MRI cases (*n* = 55), whole and central gland segmentations (*n* = 55 [327 masks])	Not reported	2.04 GB	MRI cases (NII.GZ), whole and central gland segmentations (NII.GZ)	Not reported	Not reported	Endorectal	Not reported	Segmentation uncertainty estimation	[17]
15	NCIGT-PROSTATE	BMWH	MRI (triplanar T2W)	MRI cases (*n* = 10), gland segmentations (*n* = 10)	Not reported	768 MB	MRI cases (DICOM)	3 T	GE Signa HDx	Endorectal	Not reported	Whole gland segmentation	[36]
16	PI-CAI Challenge	RUMC, UMCG, ZGT	MRI (triplanar T2W, DWI, ADC)	MRI cases (*n* = 1,500), lesions segmentations (*n* = 1,295)	2012-2021	32.5 GB	MRI cases (MHA), lesions segmentations (NII.GZ)	1.5 T (*n* = 82), 3 T (*n* = 1,418)	Siemens ([Skyra 3 T, TrioTim 3 T, Prisma 3 T, Aera 1.5 T, Avanto 1.5 T, Espree 1.5 T]; *n* = 1,221), Philips ([Ingenia 3 T, Achieva 1.5 T, Intera 1.5 T]; *n* = 279)	Phased-array surface coil	Age (*n* = 1,500), PSA (*n* = 1,460), PSA density (*n* = 1,047), Gleason grade (*n* = 1,001), prostate volume (*n* = 1,473), histopathology type (*n* = 1,001)	Disease classification, Cancer detection	[37, 38]
17	Prostate158	CUB	MRI (axial T2W, DWI, ADC)	MRI cases (*n* = 139), zones segmentation (*n* = 139), lesions segmentations (*n* = 83)	Not reported	2.6 GB	MRI cases (NII.GZ), zones and lesions segmentations (NII.GZ)	3 T	Not reported	Phased-array surface coil	Not reported	Zones segmentation, Cancer detection	[39]

*ADC* Apparent diffusion coefficient; *BIDMC* Beth Israel Deaconess Medical Center, Boston, MA, USA; *BMC* Boston Medical Center, Boston, MA, USA; *BWH* Brigham and Women’s Hospital, Boston, MA, USA; *CT* Computed Tomography; *CUB* Charité-Universitätsmedizin Berlin, Berlin, Germany; *DCE* Dynamic contrast-enhanced; *DWI* Diffusion-weighted imaging; *GE* General Electric Healthcare Systems, Milwaukee, WI, USA; *HUH* Haukeland University Hospital, Bergen, Norway; *HUP* Hospital of the University of Pennsylvania, Philadelphia, PA, USA; *LHMC* Lahey Hospital Medical Center, Burlington, MA, USA; *MRI* Magnetic resonance imaging; *NVB* Neurovascular Bundle; *PDW* Proton Density-Weighted; *PET* Positron emission tomography; *Philips* Philips Healthcare, Best, the Netherlands; *PI-RADS* Prostate Imaging-Reporting And Data System; *PSA* Prostate-specific antigen; *RUMC* Radboud University Medical Center, Nijmegen, the Netherlands; *Siemens* Siemens Healthineers, Erlangen, Germany; *SV* Seminal vesicles; *T1W* T1-weighted; *T2W* T2-Weighted; *TNCI* The National Cancer Institute, Bethesda, MD, USA; *TNM* Tumor Node Metastasis; *UCCUC* The University of California Clark Urology Center, Los Angeles, CA, USA; *UCL* University College London, London, UK; *UMCG* University Medical Center Groningen, Groningen, the Netherlands; *UPMC* University of Pittsburgh Medical Center, Pittsburgh, PA, USA; *WHSL* Washington University in St. Louis, Saint Louis, MO, USA; *WRUHC* Western Reserve University Hospitals, Cleveland, OH, USA; *ZGT* Ziekenhuis Groep Twente, Twente, the Netherlands.

Although quite some prostate MRI data seem to be available, the quality of the outcome that is to be predicted, *i.e.,* the reference standard for annotations (if any), is disputable. For datasets carrying annotations of the prostate anatomy, the reference standard is often one or few human readers (whose annotations highly depend on their experience level). Similarly, we notice that for the prediction of csPCa, the quality of annotations provided per dataset highly differ. One dataset reports pathology outcome from MRI fusion biopsies, another uses in-bore MRI biopsies, another uses radical prostatectomy, while for others the reference standard remains unclear. Accuracy across these various tissue sampling strategies can vary strongly. Inconsistencies and missing information across imaging data, cohort distribution, and reference standard can also make it difficult to consolidate multiple public datasets into one. At the same time, most public datasets are too small to be used on their own. We conclude that annotations and data characteristics for public datasets are often ill-defined, and we advise that potential users contact the data providers for additional information (*e.g.,* patient distribution, follow-up status) prior to usage.

Data overlap is an issue with public datasets. In some cases, all or part of the dataset contains cases from other public datasets. For example, the NCI-ISBI 2013 dataset [30] combined 40 cases from the Prostate-3T dataset [22] and 40 cases from the PROSTATE-DIAGNOSIS dataset [25], and the entire PROSTATEx [18] and Prostate-3T [22] datasets are included in the PI-CAI dataset [38]. Combining these datasets, may therefore inadvertently lead to false assumptions of data size in scientific AI experiments or product development.

All the datasets were confirmed to have been collected with institutional/ethical review board approval, except for I2CVB [23], Prostate158 [39], and QUBIQ21 [17], for which this information was not found. The datasets are all anonymized. Anonymization is becoming increasingly difficult in our online world in terms of data strictly not being traceable to patient information. Radiological images are almost always acquired, exchanged, and stored in DICOM format. The DICOM header is very rich in information that could lead to tracing back to the patient. The DICOM standard defines security concepts for anonymization, and public tools that implement this are available [40]. A simpler solution is to provide images in non-DICOM format, which contain header with minimal information. The drawback of non-DICOM images or very strongly anonymized DICOM images is that relevant scientific information may get lost. Public prostate MRI data should preferably be carefully anonymized DICOM images with as many tags preserved as possible. The Cancer Imaging Archive provides a very strong DICOM anonymization procedure with the most comprehensive set of DICOM tags available for scientific research [41].

Patient inclusion criteria were ambiguous for most datasets, which may raise questions about the degree of bias in the selected data. Images were mainly acquired for PCa detection in patients with suspected csPCa and/or for intervention or staging purposes. The PROSTATE-MRI [20] and Prostate Fused-MRI-Pathology [21] datasets included patients in whom biopsy confirmed cancer and who underwent radical prostatectomy. For the QIN-PROSTATE-Repeatability dataset [28], the criteria were the patient’s ability to undergo prostate MRI with an endorectal coil and complete the repeat examination. For the Medical Segmentation Decathlon (MSD) dataset [35], the criterion was the suitability for the development of a semantic segmentation algorithm.

The datasets have been used extensively by researchers for the development of clinical applications, including segmentation of prostate tissue and detection/diagnosis of csPCa [16, 42]. For segmentation-related applications, the PROMISE12 [22] and MSD [35] datasets were most commonly used for segmentation of the whole prostate gland and prostate zones, respectively (*e.g.* [43, 44]). PROSTATEx [22] is currently the dataset that is most commonly used for development of AI for detection of csPCa (*e.g.* [45–47]).

As of May 6, 2022, the PI-CAI challenge [37] has publicly released 1,500 (of 12,500) cases [38] with a much stronger reference standard than that of the PROSTATEx challenge [29]. Additionally, PI-CAI reserves a hidden testing cohort of 1,000 cases, with histopathology-confirmed positives (Gleason grade > 1) and histopathology (Gleason grade < 2) or follow-up confirmed negatives, that will span the complete distribution of patients encountered in clinical routine. Data will be multivendor (3-T scanners from Phillips and Siemens) and multicenter (Radboudumc, Ziekenhuis Groep Twente, University Medical Center Groningen, Norwegian University of Science and Technology). Patient age, PSA density, PSA level and prostate volume will be provided for all cases. Expert-derived lesion delineations are provided for approximately 80% of all cases, and AI-derived lesion delineations (pseudo-labels) are provided for all cases, using a state-of-the-art csPCa detection developed at Radboudumc [48].

An additional source of public images will be the ProCancer-I platform [49]. It was launched in 2020 to solve issues concerning national and international medical data sharing regulations, and lack of tooling, causing many institutions not to make their data available [50, 51]. To enable these institutions that are willing to share their data to improve, validate, and test state-of-the-art AI tools for prostate MRI diagnosis, the EU-funded platform provides a scalable high-performance computing platform that will host the world’s largest collection of anonymized prostate MRI image datasets (> 17,000 cases) based on data donations in compliance with EU legislation (GDPR). To ensure rapid clinical implementation of the developed models, the platform partners will closely monitor performance, accuracy and reproducibility. Optima [52] is another EU-funded initiative that aims at overcoming the limitation of data sharing while enabling research and clinical partners to leverage a variety of federated and centralized European data for the dynamic development and clinical implementation of AI tools to combat prostate, breast, and lung cancer.

Federated learning is an alternative approach to making data available. It allows to train robust prostate AI for MRI, with representative data from multiple countries and institutions, but, in contrast to the conventional approaches, in a federated learning framework, the AI model is trained by iteratively sharing model weights obtained from training on local data. Consequently, the local data need not to be shared and never leaves the hospitals [53, 54]. Promising frameworks for federated learning include Flower [55], FedML [56], and pySyft [57], which support several operating systems, the use of graphics processing units, and differential privacy. A successful example of federated training of a prostate segmentation algorithm is reported by Sarma et al. [58].

### Available AI tools for prostate MRI

An inventory of commercial/public prostate MRI AI products/tools provides an overview of available technology and supported clinical applications. This is relevant to both the clinical end user and scientists exploring knowledge gaps. In reviews, these updates quickly become outdated. Of note, a website [59] provides an updated overview of available AI-based prostate MRI software for clinical radiology.

A comprehensive overview of current commercial products/public tools is summarized in Table 2. Eleven vendors offer products that help report and acquire prostate MRI for diagnostics and intervention. The AI claims range from modest automatic segmentation of the prostate to measure prostate volume, to calculation of a tumor heatmap, up to an automated detection of csPCa [60]. Consequently, products vary in their level of clinical support and ability to improve workflow or reader variability. Only few vendors are currently able to automatically generate reports that can help reduce the diagnostic workflow. For only one vendor (Siemens Healthineers) [13], a prototype was shown to increase diagnostic accuracy and reduce variability between readers [60]. Various trials are underway, and it is expected that soon other vendors will upgrade their products with similar claims. AI for prostate MRI is not dissimilar to many other radiology applications, in that peer-reviewed evidence of effectiveness is mostly lacking [61]. As shown in Table 2, levels of certification vary, which also implies that the level of validation varies. The ‘soft’ AI engine claim of it being able to produce a tumor heatmap without explicit detection performance can do with a class I certification with little validation studies. The ability to predict presence of csPCa and associated claims of workflow improvements requires much stronger evidence and validation levels (level II and above).

**Table 2:** Overview of commercially available prostate MRI tools that implement AI. The table attempts a comprehensive comparison in terms of highest claim and level of trust based on certification level

Number	Vendor	Product(s) name	Highest AI claim	FDA	CE
1	Quibim	DWI-IVIM, DCE-PKM, Texture, T2 mapping, QP-Prostate	Quantitative MR possibly reducing machine dependence and automate some report generation	Class II	Class IIa
2	Quantib	Quantib-Prostate	Automatic tumor detection that can automate some report generation	Class II	Class IIb
3	JLK	JPC-01K	Heatmap that may help spot tumors	No	Class I
4	Siemens Healthineers	Prostate MR Syngo.via, AI-Rad companion	Automatic tumor detection that can automate some report generation	Class II	Class IIb
5	Lucida	Prostate Intelligence	Automatic tumor detection that can automate some report generation	No	Class I
6	Cortechs.ai	OnQ Prostate	Heatmap that may help spot tumors	Class II	No
7	Elekta	ABAS	Automated anatomy segmentation	Class II	Not reported
8	Mirada	DLCExpert	Automated anatomy segmentation	No	Class IIb
9	MIM	MIM Maestro	Automated anatomy segmentation	Class II	Class III
10	Philips	MRCAT Prostate + Auto-Contouring	Automated anatomy segmentation	Class II	Not reported
11	General Electric	PROView DL	Automated anatomy segmentation	Class II	Not reported

*AI* Artificial intelligence, *CE* European conformity, *FDA* Food and Drug Administration. For more information, check [59] for detailed descriptions of features and links to vendor websites and related information

Non-commercial public AI tools for prostate MRI may reflect the current state-of-the-art. Many tools have been made available in the form of publications, codes, software plugins, or grand challenge algorithms for research or non-clinical purposes. A number of these tools have already been presented in other review articles [16, 42, 62, 63]. However, access to these trained models is not always possible, and when it is possible, it is usually not easy for end users or researchers to implement. Furthermore, if developers want to benchmark against these models, they usually must use the source code, install libraries, and make changes to fit the model, which can lead to unfair and non-direct benchmarking. One way to overcome this problem is to use platforms that easily allow the use and direct benchmarking of pre-trained models.

NVIDIA Clara Imaging [64] is a platform that provides a framework for the development and direct deployment of AI applications for medical imaging. It includes a set of public, pre-trained deep learning models. Currently, the available models appear to focus primarily on segmentation tasks, including nnU-Net [43], a self-configuring method for deep learning-based segmentation that has shown excellent performance on the MSD [34] and PROMISE12 [32] challenges. Another platform is Grand Challenge–Algorithms [65], to which pre-trained models can be uploaded so that developers can directly test the method and compare their models against its performance. The platform currently includes a prostate MRI segmentation model and two csPCa detection models. Furthermore, the Federated Tumor Segmentation (FeTS) Platform [66] provides access to multiple pre-trained models that can be deployed in a federated fashion.

### Grand challenges

Grand challenges provide the means to benchmark and validate multiple AI models across a set of common training and testing datasets, in a bias-free manner. For prostate MRI, there are a handful of public challenges, each of which focus on one of two clinical outcome categories: prostate anatomy segmentation (NCI-ISBI 2013 [31], PROMISE12 [33], MSD [34], QUBIQ21 [17], Prostate158 [39]) and csPCa detection/diagnosis (PROSTATEx [29], Prostate158 [39] and PI-CAI [37]).

The NCI-ISBI 2013, MSD and Prostate158 challenges evaluated the performance of AI models for segmentation of the prostatic peripheral zone (PZ) and transitional zone (TZ). Meanwhile, the PROMISE12 challenge evaluated the segmentation of the whole gland, not its constituent zones. Segmentation of the whole gland is considered an easier task than segmentation of prostatic zones (especially PZ). This is reflected by a top Dice similarity coefficient in the literature of about 0.90 (TZ) and 0.75 (PZ) [67]. In the MSD, nnU-Net [43], which performed the best, had similar results with a Dice similarity coefficient of 0.90 and 0.77 for TZ and PZ, respectively.

PROMISE12 ranked AI-derived segmentations using a score that averages four different similarity and distance metrics relative to an expert’s manual annotations. The top score is 100, but in their article [33], challenge organizers explained that final scores are normalized with a second (inexperienced) reader to 85. They already indicated that very high scores (> 90) are likely in the realm of inter-reader variability. During the challenge, the best score achieved was 87. However, in the present-day post-challenge leader board, ten submissions have a score ranging from 89.5 to 91.9, with the highest score being achieved using MSD-Net [68]. At these higher limits of performance, differences between AI algorithms, with respect to the PROMISE12 reference standard (human expert with six years of experience), may not be indicative of better or worse performance. Particularly, with deep learning algorithms performing so well, the issue now becomes to define a better reference standard that is more representative of the biological ground-truth, which remains an open research question.

The QUBIQ21 challenge aimed to quantify uncertainties in biomedical image segmentation. Recent advances in probabilistic deep learning allow for uncertainty estimation across predictions [69], which can pave the way to explainable, trustworthy AI and can inform clinicians about diagnostic uncertainty of AI [70]. QUBIQ21 addresses multiple organs and imaging modalities, including prostate MRI. For prostate MRI, there are two segmentation tasks for 55 T2W cases (one mid-gland slice with six expert annotations per case).

The Prostate158 challenge is a recently released challenge that aims to segment the PZ and TZ of the prostate in addition to segmenting the PCa lesions. The challenge provides 139 cases for training and validation of AI models and uses a hidden test dataset of 19 cases for performance evaluation of the models.

The PROSTATEx and PI-CAI challenges aim to evaluate the performance of AI models for csPCa detection and classification. Launched in 2014, the PROSTATEx challenge has been the only public benchmark for this task to-date. More than 1,765 entries have been submitted during the challenge, with the maximum value of the area under the curve at receiver operating characteristic analysis currently at 0.95. Meanwhile, in the PI-CAI challenge provides the largest training (*n* ≈ 9,000; of which 1,500 cases will be made public), validation (*n* ≈ 100), and testing (*n* ≈ 1,000) datasets to-date, with a study design and reference standard established in conjunction with multi-disciplinary radiology, urology and AI experts in the domain. PI-CAI also includes an international reader study with 63 radiologists (42 centers, 18 countries; 1–23 years of experience reading prostate MRI, median 9 years) till-date, to assess the clinical viability of stand-alone AI relative to radiologists.

## Discussion

AI is starting to get an assistive role in the PCa clinical pathway. The advent of deep learning for medical imaging allows realizing stand-alone AI that achieves good to expert level performance in the prediction of segmentation volume and csPCa detection [14, 15, 45]. Deep learning AI models are being incorporated in products that provide human interface software that aims to help improve workflow and reduce diagnostic performance variability [45, 47, 60, 71]. Moreover, these AI diagnostic models can be used before, during and after radiation therapy. Segmentation models can be used for organ delineation in the planning phase and for prostate-targeted MRI-guided radiotherapy [72]. Detection models can for example be used to monitor the response of the lesion during and after treatment [73]. Similar developments have already been seen in other medical imaging domains such as breast [74] and lung [75]. The recent availability of prostate MRI data explains the rather late development of prostate MRI AI. The development is further complicated because prostate MRI is intrinsically multiparametric with an enormously different appearance of the image parameters.

Other complications include the presence of image artifacts [76] and that MR image acquisition is not standardized, although minimum requirements for PI-RADS reading exist [7]. An important role for AI may therefore also be in image preprocessing and quality control [77–80]. Finally, prostate MRI hampers a well-defined reference standard with definitions of cancer significance widely varying. The AI-induced large-scale collection and curation of data will help further develop the field. To that end, AI can help prostate MRI realize a better perspective for men with PCa, by reducing unnecessary biopsies, reducing overtreatment, providing early detection to achieve less burden, and increasing survival.

We have attempted to provide an overview of the current state-of-the-art of AI applications for prostate MRI. Unlike other review papers [16, 42, 62] that focus on AI tools that have been developed, this review focuses on open datasets, commercially/publicly available AI, and grand challenges. However, since this is a rapidly growing field, a limitation of this review is that it will become outdated in a relatively short period of time, just like the review papers before it. Therefore, up-to-date reviews of this field are constantly needed.

In conclusion, available prostate MRI AI products are relatively few, with only one validated for assisting in the difficult detection task and others for the simpler gland volume estimation task. The AI prediction of other clinical outcomes in the prostate cancer pathway is still maturing or even needs to start at all. A lot of research is still required to successfully realize AI to help in the whole prostate pathway. Public well-curated datasets are available but are relatively small and vary in quality of the reference standard. More computational AI challenges are needed to provide independent validation of products and research to build trust in AI for prostate MRI.

## Data Availability

Not applicable.

## Abbreviations

AI: Artificial intelligence
csPCa: Clinically significant prostate cancer
DICOM: Digital Imaging and COmmunications in Medicine
mpMRI: Multiparametric magnetic resonance imaging
MSD: Medical segmentation decathlon
PCa: Prostate cancer
PI-RADS: Prostate Imaging and Reporting and Data System
PSA: Prostate-specific antigen
PZ: Peripheral zone
TZ: Transitional zone
